# Spinal Distortions

**Published:** 1841-07-01

**Authors:** 


I.—Memoire sur les Deviations simulées de la Colonne Vertebrale et les Moyens de les distingues des Deviations Pathologiques.—Presenté a l'Academie Royale de Medicine. Par le Docteur *Jules Guérin*, Chargé du Service des Difformités a l'Hôpital des Enfans et Directeur de l'Institut Orthopédique de la Muette.

II.—Memoire sur l'Extension Sigmoide et la Flexion dans le Traitement des Deviations Latérales de l'Epine.—Lu a l'Academie Royale de Medicine.

III.—Memoire sur une Nouvelle Méthode de Traitement du Torticolis Ancien.—Presenté a l'Academie Royale des Sciences.

IV.—Memoire sur l'Etiologie Générale des Pieds-bots Congenitaux.—Lu a l'Academie Royale de Medicine.

V.—Memoire sur les Caracteres Généraux du Rachitism.—Lu a l'Academie Royale des Sciences.

VI.—The Cause and Treatment of Curvature of the Spine and Diseases of the Vertebral Column.—Illustrated with Cases and Plates. By *E. W. Tuson*, F.R.S., Surgeon to the Middlesex Hospital, &c.

The various memoirs by Doctor Guérin, the titles of which are at the head of this article, form a series of treatises on the deformities of the osseus tissue and on the remedial means to be adopted for their cure. To this subject the author has more especially devoted himself of late years, with ample opportunities for observation, and with zeal and talent well calculated to advance our knowledge.

The most voluminous of these memoirs furnishes a curious illustration of the medical history and professional economy of our neighbours, and as a fragment, " pour servir a l'histoire," as they would say themselves, is both instructive and amusing.

Amidst the literary and other extravagances of the medical profession in England, we think it would scarcely enter into the mind of a scientific surgeon, here, to devote nearly 150 closely printed pages, and eight highly finished engravings, to the discussion of the means of detecting a factitious from a real and morbid deformity of the spine.

Dr. Guérin prefaces his memoir in the following characteristic fashion:

"I will give the history of this memoir in a few words—its origin, vicissitudes, the storms it has raised, which preceded and have followed it, finally the effect, moral and scientific, which it has produced. It may be seen that this is no ordinary work, (I speak in reference to the special circumstances which have induced me to write it), but one which is attached to an epoch of my medical career—at once difficult and peculiar in its character."

This little episode of French medical politics and character, in true epic style, brings before us, in tragic order, individual and national customs and prejudices, persecutions, escapes, defeats, triumph and disaster, and winds up with a splendid peroration on the vast interests, scientific and moral, embodied in the history and involved in the results of a squabble between a medical professor and a charlatan curer of spines never deformed. This squabble, however, which shook into convulsions the Academy of Medicine and occupied the learned judges of the land in two courts of justice, is really too rich a *morçeau* to be passed over without a slight sketch for the amusement of our readers. And, indeed, this is so admirably given by the Doctor himself in the preface above quoted, that it admits of slight improvement at our hands—we shall do little more than translate.

" Some four years ago a man (not a physician) presented to the Royal Academy of Medicine several persons with curvature of the spine which he undertook to cure in the space of a few months. The Academy instituted to watch over the progress of science, and to repress charlatanism, charged a commission composed of the êlite of its members to follow the effects of the treatment adopted. After a space of about four months two of the patients passed for cured, and the commission accordingly made its report."

It chanced, however, that Dr. Guérin was apprized that one of the subjects of cure, a strong and fine girl of twenty, most extraordinarily deformed before submitted to treatment, according to the report of the commission, was particularly well made according to the account of the people among who she had lived, having previously performed all the duties of an active chamber-maid! This circumstance seemed to the Doctor very satisfactorily to explain the rapidity of a cure which he confessed he had up to that time found it difficult to understand. With this new light on the modus operandi of the man (not a physician), Dr. Guérin made some further inquiries, and began to examine the plaster casts of the cases treated —attaching himself particularly to that of Jenny Guèry (the femme-de-chambre in question). An unfortunate attachment, it appears, to all parties.

Fully convinced at last that the lady had never really been deformed—was he openly to declare his conviction to the world, or hold his peace? Difficult question—often no doubt occurring to those who are obstinately bent upon proving the object of their attention perfect. " To have been silent and let things take their course," says the Doctor, " would have been more prudent, wise, and certainly more safe, than to tell the Academy that its confidence had been abused, and to furnish the proof to that learned body that it had made so ridiculous a mistake as to cut a very sorry figure in the eye of the public,—I chose the latter." Rash man thus to provoke a respectable body of learned Doctors!

He wrote to the Academy declaring his conviction, resting as it did upon two orders of proof—the one moral, the testimony of those who knew the prior perfection of Jenny Guèry's stature; the other scientific. Dr. Guérin thought the Academy would look to the scientific evidence, instead of which it preferred the moral view, the evidence concerning which, as regarded Madlle. Jenny, was particularly contradictory and embarrassing to right-minded men. Dr. Guérin's correspondents every where heard that in her native place Jenny passed for a handsome girl—alert in intellect and perfectly well made—nay, whose " taille" was one of the best in the country! On the other hand, her curer did not not fail in testimony that the said Jenny had been cruelly deformed, of which all Paris had been persuaded when she was presented in the first instance, in fact one of the members of the Commission had christened her a "*culle de jatte.*"

Long the Assembly, in grave debate, agitated this most complicated question, each party restricting themselves to the moral testimony which, being both black and white, was especially embarrassing. At last the Academy found out that with respect to Jenny there were not sufficient proofs before it that she had simulated the deformity or, consequently, deceived so grave and sagacious a body; and Dr. Guérin discovered that he had forgotten to allow for the little weaknesses and passions of man, even in a learned assembly, and that in the revelations which he had made, he had compromised in their own eyes, their gravity, wisdom, and infallibility; they were not neutral arbitrators and judges but partizans against him. Strange that he should never have read the pithy sentences of his countryman, La Rochefoucault, and better appreciated the influence of man's "*amourpropre.*" The Professor, however, hoped to be quit of all further ill consequences of his rashness at the price of this partial check—alas!

> "Troubles do never come in single files,
> But in battalions ever!"

And Jenny and her friends, inspired by this triumph, now assailed the Professor in a court of justice for defamation of character—and gained their cause. They failed, however, in either tiring or convincing their detractor, and he appealed to the higher tribunal of the *Cour Royale;* at the same time that he once more agitated the subject in the Academy, bringing forward scientific proofs of the absence of all morbid deformity. The plot thus thickened and a double action was carried on. In the first trial the decision of the Academy had been quoted as confirming the propriety of the sentence—but now that the same learned body saw the error of their ways and confessed they had been deceived, it was quoted in the Cour Royale as a strong reason for the Doctor's conviction. The first sentence therefore was confirmed, and M. Guérin condemned in costs and fined—as a "*Defamateur.*"

Dr. G. at this stage made certain discoveries calculated to prove of great utility to all professional writers who trust to be held harmless under the shield of truth—if they would but profit by other people's experience without insisting on paying for it. These discoveries must be classed under the heads of moral and legal facts elicited in this momentous affair.

1. The law does not jest. Dr. Guérin had indulged, he tells us, in certain little pleasantries, and among others in that of following Boildeau's example and calling things by their proper names. Not finding any more accurate term by which to designate a man practising medicine in the market-place without a diploma, than *Charlatan,* he adopted it, and rendered himself decidedly guilty of the crime of abusive language.

2. Since 1819, in France a law exists not sufficiently known: by this law he who publicly imputes to a citizen a reprehensible action, whatever be the proofs, is punishable as a defamer. Now defamation legally is defined to be "any allegation which injuriously affects the honor or consideration of another,"—of course the more true the allegation the more surely injurious—the more certainly do you merit condemnation. These things and many others Dr. Guérin assures the world his treatise on the nature and treatment of simulated spinal distortion taught him—but, hélas, too late to be useful!

And here endeth this eventful history—thus wound up by the Doctor:—

"From the historical part of my memoir let us turn to its scientific aspect. Persons who have endeavoured to diminish the importance and authority of my researches have sought to reduce its proportions into that of a mere personal discussion. But independent of its value as a work of critical analyses, it embodies new facts in anatomy, physiology, and pathology, which give it a character purely scientific, &c."

For these facts we refer our readers to the memoir itself, and should they ever feel inclined to call things by their proper names, obviously a very reprehensible practice, or be anxious to learn how to distinguish fictitious from morbid distortions of the spine, we strongly recommend their perusal of Dr. Guérin's Memoirs. It contains useful matter, notwithstanding that its readers may be, like ourselves, tempted to smile at its pleasant admixture of the grave and ludicrous, and of grandiloquent deductions from very Liliputian facts—telling the oft-told story,

"How mighty contests rise from trivial things!"

We pass on to the other memoirs which are both more scientific in character and of more practical value, and first to the treatise on lateral curvature of the spine. We have also to draw attention to Mr. Tuson's volume on the same subject.

On taking up the latter work we were for some time at a loss to divine for what particular class of readers Mr. T. had designed his observations. Whether for nurses, mothers, students, practitioners, the lay public generally, or, finally, for each and all together. The matter and the style of the first 100 pages seemed but little adapted to any but the two first, and not the most sensible of these. Commencing at the earliest possible causes that might influence the growth and curvative of the spine, Mr. Tuson objects rationally enough to swathing with tight rollers the body of the new born infant, but no ordinary phraseology suffices for the purpose, it is not enough to say that no advantage, but much injury may arise from it. Mr. Tuson startles the nurse or the mother, whichever it may be, into conviction, by declaring it a practice,

"Which, being contrary to the laws of nature, and tending to obstruct her power and molest her laws, stands self-condemned!"

The author, however, improves upon acquaintance, and seems more clearly to address himself to the profession as he advances in his subject. He has evidently paid considerable attention to the subject and enjoyed fair opportunities of observation. The great object of the work to which the author very frequently recurs, would seem to be the necessity for ascertaining the *cause* of any deviation from the natural form in the spine, prior to determining the treatment for its removal. This is a principle so clear and trite that we scarcely should deem its enforcement required by any educated medical man, who to some learning could add a little common sense. Mr. T., however, evidently thinks otherwise; he tells us—

"There are works on affections of the spine, whose authors lay down and recommend *one* general plan of treatment, whatever be the cause of the complaint; whether scrofula, general debility, caries, or a softening of the bone,—whether the curvature be lateral, or in any other direction,—even angular projection is not exempted,—but one mode of cure is to be followed." 70.

And again, in reference to the labours of others in the same department, we are told—

"It is only of late years that scientific men—from some strange hallucination—have turned their attention to this disease; but all the treatises hitherto published —however excellent they may be when applied to specific cases—appear to me to partake more or less of theoretical reasoning. A disease presenting itself under so many different features, with the same characteristics, must require a specific treatment, adapted to each form it assumes: a mechanical contrivance, therefore, that would act with advantage in curing a deformity offering certain peculiarities, would have a diametrically opposite effect when it showed itself with other combinations. Hence it follows that *any one plan of treatment for all cases indiscriminately* must be fallacious. This palpable error struck me most forcibly on my first commencing my professional career, and time and observation have contributed to confirm it. I felt there was a large field for investigation,—a wide chasm that might be advantageously filled up."—*Preface*, p. x.

"Various plans have been recommended to remedy curvature of the spine, and the author of each has pertinaciously adhered to his own, treating all cases indiscriminately, and not looking, as should be done, to the cause of the curvature, and whether it be in a lateral or any other direction. Extension, rest, counter-irritation, instruments, pressure, exercise, &c. have each been recommended and used by their own advocates." 74.

This we cannot believe to be a very accurate statement of the practice or state of the profession as regards spinal disease, and we think does less than justice to it, and to the authors whom he himself quotes.

"But while I thus expose the ignorance of the empiric, let it not be supposed I condemn the works of those scientific men who have devoted their attention to the subject, and to whom the profession and the public are much indebted for useful and scientific observations on the affections and diseases of the spine. I submit the names of Sir B. Brodie, Earle, Shaw, Bradly, Bampfield, Thompson, and others; but I am not aware that these gentlemen have ventured to introduce or adopt any single mode or plan of treatment as applicable to all cases. Sir B. Brodie's observations on caries of the spine are well entitled to the attention of every practitioner. Earle's are scientific, and the means recommended by him useful, mechanical, and ingenious. The work of my departed friend Mr. John Shaw, will be found useful to all those who are desirous of better acquaintance with this subject: his plan of treatment was employed while I held the office of house-surgeon to the Middlesex Hospital, in several cases, with beneficial result. Bradly's observations are worthy of attention. Bampfield's work is interesting and instructive. Thompson's remarks may be read with attention, and his treatment may prove useful and advantageous *in some cases*." 75.

This is dismissing the claims of these gentlemen rather summarily, but yet proves that, as far as the *profession* is concerned, scientific men *have* given their attention to the subject, and that the pressing danger which Mr. Tuson seems to have very constantly before him of medical men treating spinal cases empirically—upon one system, without reference to the great diversity of causes, not less than differences in the natnre of the disease resulting, is by no means apparent.

Dr. Guérin's able treatises on this subject shew that spinal diseases and deformities are studied with all the advantages which the present state of our general and professional knowledge afford. He tells us that his memoir is intended to make known a new method of effecting the straightening of lateral deviations of the spine by lateral or sigmoide extension, and by flexion—a mode based upon two new principles which are substituted for two old ones—oblique or perpendicular extension substituted for parallel—and flexion instead of compression. These principles, which he has here first applied to curvatures of the spine, are applicable to the treatment of all articular deformities.

In addition to this, the memoir contains certain general principles from which, in his other treatises, the mechanical treatment to be adopted for other deformities of bone are deduced.

Since experience has shown the insufficiency of medical means and the indispensable necessity of mechanical agents in the treatment of lateral deviations of the spine, a great number of machines have been proposed and adopted—all with a view to produce extension of the spine in its long axis, or in other words parallel, and pressure upon the convex portions of the deformities. This was first attempted by means of portative machinery or corsets resting on the hips—subsequently extension in a horizontal direction was substituted, the patients being placed on variously-contrived couches while the force in opposing senses was applied—and with this view, up to the present time, have all the various kinds of extending apparatus been devised. Thus, in two words, the mechanical treatment has been confined to *parallel extension* and to *lateral pressure*.

Notwithstanding many cases of failure from the indiscriminate and injudicious use of these means, enough benefit has resulted to leave no doubt as to the utility of mechanical treatment.

M. Guérin thus proceeds to explain his mode of mechanical treatment, and the principles on which it is founded.

"What have we in view to accomplish in the treatment of lateral curvatures of the spine in reference to mechanical agents? To straighten a stem curved in its length at one or more points. If this be given as a problem for solution, stripped of all organic circumstances, which only serve to obscure and hide its simplicity, and it be stated simply as a stem curved or bent, and to be made straight, there is no man even of mediocre intelligence who, with merely the benefit of common experience, will not present a solution of this question infinitely more satisfactory than all those hitherto proposed as a means of cure for a curved spine. What would he do in fact? In place of the spine put a flexible stem, of any material which is curved, into his hands—assuredly he would not commence by taking hold of the two ends and pulling in the direction of its length. With each hand he would fix the extremities and press the convexity of the curve against his knee—he would pull perpendicularly on each of the ends and produce a curve in the sense opposed to the only previously existing. He would not be content with stretching it until the first curve disappeared, because he would know by experience that, to obtain a complete and permanent straightening, he must go further, and make a curve in the opposite direction, in order to overcome the force which tends to reproduce the curve when we limit our efforts to merely bringing it to a straight line.

Thus would any one proceed in order to straighten any kind of curve in a flexible stem, and this is what I have sought to reduce to practice for the treatment of curvatures of the spine. The method which I have proposed consists in substituting artificial curvature in a sense directly opposed to those produced pathologically, so as to give to the vertebral column the form of an S in the reverse direction to that which the diseased curvature presents—in other words, it consists in substituting oblique and perpendicular for parallel extension of the spine, which I shall call the *sigmoid* extension, the mere name sufficing to indicate the object proposed and which, I believe, to be realised in the apparatus I am about to describe."

This apparatus we will not attempt to describe in all its detail, but refer our readers to the memoir itself; suffice it to say that it is constructed with a view to produce at the same time flexion and extension of the spine, thus greatly diminishing the *degree* of extension, otherwise required to conquer the elastic force of the muscular and ligamentous structure, which tends to reproduce the curve when the extension has ceased. The increased extension required, when alone employed, more especially when the spine is reduced nearly to its proper line, is followed by many evil consequences—the fibro-cartilaginous, ligamentous, and muscular structures corresponding to the concavity of the curves, are inordinately stretched and weakened, the antero-posterior curves, which are natural to a healthy spine, are obliterated, though of great importance to the solidity and natural figure of the trunk.

This, observes Dr. Guérin, is so true that the greater number of persons who leave the institutions where the bed of Wurtzbourg is exclusively employed, are flattened in the back—the shoulder-blades and different regions of the vertebral column being all on the same plane, destroying the natural grace and contour of the figure. A still more grave inconvenience results from the parallel extension upon the spine—separating the vertebræ from each other, in order to obliterate the curvature, has no action or tendency whatever to reduce the superabundant development of one half of the fibro-cartilage and bodies of the vertebræ corresponding to the convexity.

That such is the state of parts Dr. Guérin quotes the opinion of M. Ribes, supported by several pathological specimens, proving the impossibility of obtaining in any very short period a total cure of these lateral curvatures of the spine.

"It is only necessary," says M. Ribes, "to cast the eye upon these preparations to feel convinced that methodical extension, judiciously applied and long continued, can alone re-establish the natural condition of the parts—and, in order to produce this, it is necessary that the means adopted *should press the bodies of the verbetræ the one upon the other, on the convex side of the curvature,* so that, in pressing down on the one side, the compression may be diminished towards the concavity, and the morbidly thickened part of the vertebræ may gradually diminish while the vertebral column acquires its natural position."

This result is not to be obtained by mere parallel extension—that is in the long axis of the spine, and thus we may account for the constant tendencies to speedy reproduction of curvatures, even when apparently altogether removed by this kind of extension; the bodies of the vertebræ still remaining depressed on the sides where concavity had existed, below the level of the thickened bodies and fibro-cartilages on the convex.

Dr. Guérin states that extension in some cases is altogether inapplicable and only flexion should be employed, for which the apparatus he describes is equally adapted. These cases he defines to be those where four curves exist, or several in the same region, or two curves in the dorsal region, or curvatures too near to each other in any part of the spine.

In January, 1836, Dr. Guérin commenced the treatment of fourteen cases, all young subjects under the observation of a Commission of the Academy of Medicine. Their report of the result at the end of fourteen months is highly satisfactory.

In this treatise M. Guérin has confined himself exclusively to the best means of the mechanical treatment.

Mr. Tuson enters at considerable length into the causes of various curvatures and the general treatment they require. He divides the subject of his work into—1, Diseases of the vertebral column producing curvature and deformity of the chest, trunk, and pelvis; 2, Diseases of the column unattended by this result; 3, Injuries of the column and their results. Under the first head he takes care to draw attention to the fact, that deformity of the spine by no means necessarily implies disease in the column, since it may be a result of morbid affections of any other part of the body, as for instance—disease in the hip, knees, ancle, foot, or a shortening of either leg—these cases he terms "secondary lateral curvature," and the distinction is obviously one of great importance. We do not observe much deviation in the views he advocates from those common to the profession, except in his decided objection to the continued recumbent position; his reasons for a contrary practice are good, and seconded as they are by considerable experience, we have no hesitation in strongly recommending these opinions to the attention of our readers.

"To keep the patient constantly in the recumbent position is wrong, inasmuch as it prevents the muscles from undergoing the slightest degree of action, subjecting the joints, not only of the spine, but of the extremities, to become weak and stiffened. A physician of considerable eminence mentioned to me, whilst engaged in writing on the present subject, that he had been called professionally to see a patient who had been under the treatment of Dr. Harrison for four years, and who had been kept constantly in the recumbent position during the whole of that period. The consequence was, that the want of muscular action had deprived her of the use of her limbs. When an attempt was made to place her in a sitting posture, the joints were found to be stiff and incapable of flexion. She was doomed, therefore, to remain for the rest of her life upon a couch, perfectly helpless, and requiring constant assistance." 78.

"In regard to Dr. Harrison's plan of keeping his patient constantly in the recumbent posture, the benefits to be derived from it are that the whole spine, and very numerous muscles attached to it, are kept constantly in a state of rest and inaction; that the weight of the trunk does not press upon any of the bones of the spinal column; and that the whole weight of the head and arms is removed from the spine. So far I speak favourably of the recumbent position; but in all cases where the contrary is practicable, I would rather dispense with constant recumbency, and for the following reason, namely, that the general health of the patient is of the utmost importance, and that the functions of life cannot possibly be carried on properly when the patient is constantly lying on the back, and without exercise. Nature being thus unassisted, the muscles which beneficially press against the various viscera and other important organs of the body, and excite them to the performance of the functions of secretion, digestion, and sensation, are not brought into action. In many cases the constant recumbent position must of necessity be had recourse to; as when a patient, in consequence of the rapid increase of affection of the spine, loses the use of the limbs, or where the limbs become so weak and debilitated as to be incapable of motion; and such cases are by no means uncommon." 86.

The author is equally opposed to the late Dr. Harrison's system of treatment as regards pressure.

"Dr. Harrison, during the time of employing extension of the spine, was accustomed to make pressure against the spinous and transverse processes, with an instrument flat at its lower part placed against them, and also against the oblique processes commencing at the upper, and gradually passing down the spine to its lower part: the curve being towards the right, his object was to push the bones towards the left side; and the lower curve being in the opposite direction, he endeavoured to push the bones straight, asking the patient if she did not find the bones yield to the pressure he employed.

There are great objections to this plan of making pressure upon the spine during the time of extension. In the first place, it must be obvious that the spine being pummelled and kneaded for an hour at a time, must run great risk of being injuriously affected, without the probability of gaining any ultimate good. Such pressure must on the contrary produce considerable irritation, by bruising the attachments of the muscles; and by causing their fibres to retract, it will only draw the bones out of their places. Whereas, if the muscles are kept in a quiescent state, and free from action, they would prevent the vertebræ from being displaced. The plan of extension is, I am fully persuaded, of the greatest benefit; but it must be lessened by pressure being simultaneously made upon the spine. It may be said that pressure against the muscles will relax their fibres, but then it ought to be made against the belly of the muscle, and not against their attachment to the bones." 82.

The following observation is judicious:—

"Gymnastic exercises are strongly advised by some practitioners, in addition to the use of artificial support, by which means the muscular structure in general will acquire increased power and strength; but if the spinal column be in a weakened or attenuated state, such exercise will, by placing the weight of the various parts of the body upon the spinal column, only increase the curve. If, on the contrary, such exercises be used when the weight of the frame is removed from the spinal column, the greatest benefit will be obtained, as will be hereafter pointed out." 91.

Mr. Tuson has devised a number of exercises which may be performed in the facial or dorsal recumbent position on the couch he is in the habit of using, tending to expand the chest and improve the tone of the muscular system; he says—

"The muscular system takes an active part in curing, or in causing affections of the spine to increase; and we must be very mindful in our treatment not to lose sight of this very important fact, for where in some cases the action caused by the contending power of opposite muscles will strengthen and invigorate the surrounding parts, and excite a healthy osseous secretion, so in others the conflicting power of the stronger muscle over its antagonist, will only displace the bone, increase the curve, and produce incalculable mischief." 135.

We pass over the second division of Mr. Tuson's work, wherein he treats of those diseased states of the spine, which are unaccompanied by deformity, to make a few observations on the chapter devoted to injuries of the spine, and certain operations, confining himself to those injuries where the spinal column itself is injured—leaving unnoticed injuries and other affections of the spinal marrow, a subject which he justly observes is full of physiological interest.

Mr. Tuson remarks that sometimes the arches of one or more of the vertebræ may be fractured and the bone depressed, so as to cause pressure on the cord, and as these accidents have proved fatal, he thinks it desirable to follow the example of the late Mr. Cline and Mr. Tyrrel, and treat them on the same principle as fractures of the cranium with depression. The cases are not quite analagous—the arch of the spine is deeply covered and considerable violence and disturbance must attend any such operation; the two cases in which this method was attempted were both fatal. We should be inclined to say that under these circumstances, and with the present uncertainty as to the signs of *concussion* of the spinal cord as distinguished from its compression, the operation should not be lightly undertaken nor indiscriminately adopted in every case of paralysis from injury of the spine, otherwise many cases, which now ultimately do well, are likely not only to be unnecessarily subjected to a more or less painful and dangerous operation, but placed under unfavourable circumstances for recovery. "Modern surgery," which Mr. T. says, "amidst all its improvements has left this accident to take its chance, without lending a helping hand to assist nature in remedying the injury," may have exercised a very sound discretion which is not only the better part of valour, but of surgery also. The mania of the present day is to meddle, to lend what is called a "helping hand," which often proves anything but beneficial to the patient.

Mr. Tuson next proceeds to consider the subcutaneous division of the muscles, or their tendons, for the cure of spinal curvature, and here he is somewhat more cautious in his opinion—he says :—

"One fixed rule, however, I may venture to lay down, which is, that the operation must be confined to cases of simple lateral curvature, where the disease is entirely owing to the undue action of muscles, and where the spinal column is not the seat of disease; for it must appear evident, that where the bones of the spine are diseased in any way, the simple division of a set of muscles would only give an ascendant power to their opponents, and produce deformity in another direction. Again, it is not all cases indiscriminately of simple lateral curvature that may be selected as fit subjects for operation; a healthy diathesis is an essential and indispensable requisite; for if there is any constitutional disease, such as scrofula, &c., the use of the knife may be productive of the very worst consequences." 271.

Two of M. Guérin's memoirs are devoted to the subcutaneous operations for the division of muscles—one for confirmed wry-neck, and the other for club feet.

In reference to the operation for wry-neck, described in this memoir, the author informs us, that it rests upon two facts of pathological anatomy, which he believes to be new :—

1. In wry-neck the contraction and arrested muscular development are for the most part exclusively limited to one of the two heads or divisions of the sterno-cleido-mastoid muscle, and most frequently the sternal division, which he considers anatomically and physiologically a muscle distinct from the cleido-mastoideus or clavicular division.

2. There is in this deformity, independent of the inclination of the head on the side of the retracted muscle, an inclination in an opposite sense of the cervical column on the dorsal region, which invariably continues after the section of the muscle.

These two facts led him, on the one hand, to simplify the surgical operation by section of the contracted muscular fibres of the sternal portion only, and this by means of a simple puncture through the skin: and on the other, by the invention of a machine, or apparatus, calculated by consecutive treatment to restore the position of the head and remove the cervico-dorsal curvature. This mechanical and consecutive treatment being as indispensable for the attainment of a cure as the division of the contracted muscular fibres by the knife. To effect the former, Dr. G. has invented what he terms an orthopèdic couch—into the description of which we will not however enter.

How far Dr. Guérin has the merit of first proposing and performing the subcutaneous operation for these cases of the muscle, and in the majority of cases of the *sternal* division only, it is needless here, at least, to discuss at any length; the improvement, doubtless, is most important, and the consecutive mechanical treatment, reduced as it were to a system by Dr. G., is no less obviously an essential element of cure. The methods in practice before the mode indicated by Dr. Guérin were not only unsightly and dangerous in their results, but ineffective.

In 1822, it appears, M. Dupuytren led the way to this improved method of operation, differing only in this, that he passed the point of the bistoury through on the opposite side of the muscle, and sawed through the fibres as it were without dividing the skin above. Stromeyer, later, with a similar view of saving the skin, pinched up a fold of skin parallel with the muscle, and, pushing a bistoury through the base of this fold, divided the muscle from its anterior to its posterior surface, making only two small incisions through the skin. Finally, in 1832, Mr. Syme, in Edinburgh, performed a similar operation apparently much in the same manner as that adopted later by Dr. Guérin with a single puncture.

The memoir on congenital club foot contains some interesting researches as to its causes, tending to prove that this deformity results from convulsive muscular contractions in the fœtus, and depend upon an abnormal state of the cerebro-spinal nervous system—producing first, a shortening or contraction of the muscles of the leg or foot; secondly, a certain degree of paralysis; and, thirdly, an arrest of all consecutive development, which prevents their proportionate increase with the accompanying enlargement of the bones, and thus during the growth of the skeleton continually increasing the deformity.

We must defer any observations on the memoir devoted to Rickets, and conclude with an assurance to our readers that these treatises of Dr. Guérin's will amply repay perusal.

